# Cognitive Fatigue Is Associated with Altered Functional Connectivity in Interoceptive and Reward Pathways in Multiple Sclerosis

**DOI:** 10.3390/diagnostics10110930

**Published:** 2020-11-10

**Authors:** Michelle H. Chen, John DeLuca, Helen M. Genova, Bing Yao, Glenn R. Wylie

**Affiliations:** 1 Kessler Foundation, East Hanover, NJ 07936, USA; mchen@kesslerfoundation.org (M.H.C.); jdeluca@kesslerfoundation.org (J.D.); hgenova@kesslerfoundation.org (H.M.G.); byao@kesslerfoundation.org (B.Y.); 2 Department of Physical Medicine and Rehabilitation, New Jersey Medical School, Rutgers University, Newark, NJ 07103, USA

**Keywords:** multiple sclerosis, functional connectivity, fatigue, neuroimaging, fMRI

## Abstract

Cognitive fatigue is common and debilitating among persons with multiple sclerosis (pwMS). Neural mechanisms underlying fatigue are not well understood, which results in lack of adequate treatment. The current study examined cognitive fatigue-related functional connectivity among 26 pwMS and 14 demographically matched healthy controls (HCs). Participants underwent functional magnetic resonance imaging (fMRI) scanning while performing a working memory task (n-back), with two conditions: one with higher cognitive load (2-back) to induce fatigue and one with lower cognitive load (0-back) as a control condition. Task-independent residual functional connectivity was assessed, with seeds in brain regions previously implicated in cognitive fatigue (dorsolateral prefrontal cortex (DLPFC), ventromedial prefrontal cortex (vmPFC), dorsal anterior cingulate cortex (dACC), insula, and striatum). Cognitive fatigue was measured using the Visual Analogue Scale of Fatigue (VAS-F). Results indicated that as VAS-F scores increased, HCs showed increased residual functional connectivity between the striatum and the vmPFC (crucial in reward processing) during the 2-back condition compared to the 0-back condition. In contrast, pwMS displayed increased residual functional connectivity from interoceptive hubs—the insula and the dACC—to the striatum. In conclusion, pwMS showed a hyperconnectivity within the interoceptive network and disconnection within the reward circuitry when experiencing cognitive fatigue.

## 1. Introduction

Fatigue is one of the most prevalent symptoms of multiple sclerosis (MS) [1,2], an immune-mediated, neurodegenerative disorder characterized with demyelination, axonal injury, and brain atrophy. Fatigue is a major clinical concern among persons with MS (pwMS), as it significantly disrupts functional independence and quality of life [3]. A large-scale, retrospective study of the New York State MS Consortium registry (*n* = 5428) found that baseline moderate to severe fatigue was a significant predictor of decline in sustained neurologic disability and psychosocial limitations four years later [4]. Despite its high prevalence and debilitating impact on pwMS, the pathophysiology of fatigue is still not well understood, which results in limited effective treatments for fatigue in MS [5]. Therefore, it is imperative to understand the mechanisms underlying fatigue, in order to develop effective treatments for fatigue and improve the lives of pwMS.

One method to explore the mechanisms underlying fatigue is to utilize neuroimaging. Seminal work by Chaudhuri and Behan [6] highlighted the basal ganglia’s role in central fatigue, which was defined as “the failure to initiate and/or sustain attentional tasks (‘mental fatigue’) and physical activities (‘physical fatigue’) requiring self-motivation.” Central fatigue is common in central nervous system disorders such as MS and is qualitatively different from peripheral fatigue, which is more characteristic of neuromuscular conditions such as myasthenia gravis. Peripheral fatigue primarily affects physical activity such as exercise, sparing mental processes. There has been a body of literature using neuroimaging to confirm the presence of central fatigue [7]. The current study examines cognitive (or mental) fatigue, which is a type of central fatigue. Consistent with Chaudhuri and Behan [6]’s model, several investigators have supported the hypothesis that cognitive fatigue is associated with abnormalities in the cortical-striatal network, including the striatum of the basal ganglia, the thalamus, and the ventromedial prefrontal cortex (vmPFC) [7,8,9]. Specifically, it is hypothesized that one’s effort toward a task depends on the perceived reward from performing the task. In MS, it is suggested that there is a mismatch between the perceived effort required for a task and the resulting benefit due to abnormal reward processing in the cortico-striatal circuitry, which may lead to the experience of cognitive fatigue [8].

Furthermore, recent evidence has suggested a link between fatigue and deficits in interoception, or self-awareness of bodily internal states [10,11,12]. According to this framework, the subjective feeling of fatigue may reflect the brain’s metacognitive interpretation of the body’s failure to control internal states, due to disruptions in the interoceptive pathways. Consistent with this hypothesis, Gonzalez Campo and colleagues [13] empirically identified interoceptive deficits among fatigued pwMS, along with decreased gray matter volume and increased functional connectivity in the insula and the anterior cingulate cortex (ACC)—both key regions involved in interoception; these behavioral and brain alterations were not present in non-fatigued pwMS or healthy controls (HCs). Supporting both reward processing and interoceptive hypotheses, a motivational fatigue network consisting of the dorsal ACC (dACC), the dorsolateral prefrontal cortex (DLPFC), the vmPFC, and the insula have been proposed in recent reviews [12,14]. This network monitors the internal bodily states and chooses whether to continue exerting effort based on the perceived value of such effort (i.e., whether the effort is “worth it”). Using this framework, the level of fatigue increases as effort is expended, and the perceived value of effort declines; effort previously associated with high value becomes less rewarding, which leads to decrements in motivation and task performance.

We previously demonstrated that cognitive fatigue may be the result of inefficient cerebral activation when meeting increased task demands [15]. However, our previous study examined activations of isolated brain regions without accounting for the inter-connections between them, which precluded us from being able to implicate specific brain networks (and thus mechanisms) involved in fatigue. To investigate neural mechanisms underlying MS-related cognitive fatigue, the current study assessed functional connectivity among key brain regions underlying reward processing and interoception. Participants underwent functional magnetic resonance imaging (fMRI) scanning while performing a working memory task (n-back), which consisted of two conditions—one with higher cognitive load (2-back) to induce fatigue, and one with lower cognitive load (0-back) as a control condition. Task-independent functional connectivity in the error (residual) term was used to isolate fatigue-related connectivity. We hypothesized that pwMS would exhibit alterations in task-independent functional connectivity within reward processing and interoceptive networks in the fatigue-inducing 2-back condition compared to the 0-back control condition, compared to demographically matched HCs.

## 2. Materials and Methods

### 2.1. Participants

PwMS and HCs were recruited from the community by advertisements and word of mouth. All participants were between the ages of 18 and 65 years, right-handed, and fluent English speakers. Exclusionary criteria included: history of neurologic disorders (other than MS), serious mental illnesses (e.g., bipolar disorder, schizophrenia), substance use disorders, or learning disabilities; current use of steroids, benzodiazepines, or neuroleptics; and MS exacerbation/relapse within the past month. All participants were screened for MRI contraindications (e.g., metals in body, medical contraindications as determined by a physician, claustrophobia, pregnancy). All participants provided written informed consent before enrollment. The study was conducted in accordance with the Declaration of Helsinki, and all study procedures were approved by the Kessler Foundation institutional review board (IRB number: R-663-10; 15 May 2010).

### 2.2. Procedures

Before the experimental paradigm, examples of mental fatigue were explained to the subjects, and subjects were informed that they would be required to rate their level of mental fatigue at times throughout the scan. All participants underwent fMRI scanning while performing a working memory (n-back) task. They were asked to report their levels of mental fatigue before and after each run (i.e., fMRI block).

### 2.3. N-Back Task Paradigm

The n-back working memory task was administered using the E-Prime software [16]. All stimuli were presented on a screen located at the back of the magnet bore (a back-projection system was used) that subjects viewed via a mirror that was attached to the head coil directly in their line of sight. Subjects responded using a two-button button box (Cedrus Corp., San Pedro, CA, USA) with their right hand. There were two conditions to the n-back task: a 0-back condition, designating a low working memory load, and a 2-back condition, indicative of a higher working memory load. For the 0-back condition, participants were required to press a button with their index finger when the target letter “K” appeared on the screen. For the 2-back condition, participants were required to press the same button if the target letter was identical to the letter two trials prior in the sequence. All participants practiced both conditions prior to the scanning session. Each condition consisted of four runs (eight runs in total), with 65 trials per run. All four runs of each condition were presented in a block, followed by the four runs of the other condition. The order of the conditions was counterbalanced.

Task stimuli were in white and Arial 72-point font, with a black background. We chose 17 of the 26 English letters that have optimal discriminability from each other. The final stimuli set consisted of A, B, C, D, F, H, J, K, M, N, P, Q, R, S, T, V, and Z. All letters were presented with equal frequency. Each letter stimulus was shown for 1.5 s (and was not removed when participants responded), followed by a 500 millisecond inter-trial interval. Each run lasted for 260 s. We used the Optseq2 program (part of FreeSurfer; https://surfer.nmr.mgh.harvard.edu/optseq/) to jitter the interval between successive trials, in order to deconvolve the data as an event related design. This was accomplished by inserting six null events (duration was a multiple of the length of the trial) between successive trials. The average inter-trial interval was 1587.87 milliseconds, and the standard deviation was 1769.7 milliseconds. Accuracy and response time were dependent variables.

### 2.4. Fatigue Assessment

The Visual Analogue Scale of Fatigue (VAS-F) [17] was used to assess the level of state mental fatigue. Participants were required to report their level of mental fatigue “right now, at this moment”, on a scale from 0 (“not fatigued at all”) to 100 (“the most fatigue imaginable”), between fMRI runs. There were five VAS-F ratings per n-back task condition (0-back and 2-back): one before the first run of each condition and one after each of the four runs. We also asked participants to report levels of happiness, sadness, pain, tension, and anger (which were not analyzed), to mask the purpose of the study. 

### 2.5. Neuroimaging Acquisition

Participants were scanned on a 3T Siemens (Malvern, Pennsylvania, USA) Allegra scanner. Functional fMRI data was collected using a T2*-weighted pulse sequence (repetition time (TR) = 2 s; echo time (TE) = 30 ms; flip angle = 80; field of view (FOV) = 220 mm; slice thickness = 4mm; number of slices = 32; in-plane spatial resolution = 3.4 × 3.4 mm^2^, matrix = 64 × 64). For each run of the two n-back task conditions, 140 images were acquired. A T1-weighted image was also acquired (TE = 4.38 ms, TR = 2 s, FOV = 220 mm, flip angle = 8°, slice thickness = 1 mm, NEX = 1, matrix = 256 × 256, in plane resolution = 0.86 × 0.86 mm^2^) for functional localization and normalization into standard Montreal Neurological Institute (MNI) space.

### 2.6. Neuroimaging Data Processing

The neuroimaging data was preprocessed using fMRIPrep version 1.4.1 [18] (RRID:SCR_016216), which is based on Nipype v. 1.2.0 [19] (RRID:SCR_002502). For anatomical preprocessing, the T1-weighted (T1w) image from each subject was corrected for intensity non-uniformity (INU) with N4BiasFieldCorrection [20], distributed with ANTs v. 2.2.0 [21] (RRID:SCR_004757), and used as T1w-reference throughout the workflow. The T1w-reference was then skull-stripped with a Nipype implementation of the antsBrainExtraction.sh workflow (from ANTs), using OASIS30ANTs as target template. Brain tissue segmentation of cerebrospinal fluid (CSF), white-matter (WM) and gray-matter (GM) was performed on the brain-extracted T1w using fast [22] (FSL 5.0.9; RRID:SCR_002823). Volume-based spatial normalization to one standard space (MNI152NLin2009cAsym) was performed through nonlinear registration with antsRegistration (ANTs v. 2.2.0), using brain-extracted versions of both T1w reference and the T1w template. The following template was selected for spatial normalization: ICBM 152 Nonlinear Asymmetrical template v. 2009c [23] (RRID:SCR_008796; TemplateFlow ID: MNI152NLin2009cAsym).

For functional data preprocessing, the following preprocessing was performed on each of the eight BOLD runs of fMRI data per subject (i.e., four runs of each condition). First, a reference volume and its skull-stripped version were generated using a custom methodology of fMRIPrep. The BOLD reference was then co-registered to the T1w reference using flirt [24] (FSL 5.0.9) with the boundary-based registration [25] cost-function. Co-registration was configured with nine degrees of freedom to account for distortions remaining in the BOLD reference. Head-motion parameters with respect to the BOLD reference (transformation matrices, and six corresponding rotation and translation parameters) were estimated before any spatiotemporal filtering using mcflirt [26] (FSL 5.0.9). BOLD runs were slice-time corrected using 3dTshift from AFNI (National Institute of Mental Health, Bethesda, MD, USA) 20160207 [27] (RRID:SCR_005927). The BOLD time-series (including slice-timing correction when applied) were resampled onto their original, native space by applying a single, composite transform to correct for head-motion and susceptibility distortions. These resampled BOLD time-series will be referred to as preprocessed BOLD in original space or just preprocessed BOLD. The BOLD time-series were resampled into standard space, generating a preprocessed BOLD run in (MNI152NLin2009cAsym) space. First, a reference volume and its skull-stripped version were generated using a custom methodology of fMRIPrep. Several confounding time-series were calculated based on the preprocessed BOLD: framewise displacement (FD), DVARS (the spatial root mean square of the data after temporal differencing), and three region-wise global signals. FD and DVARS were calculated for each functional run, both using their implementations in Nipype (following the definitions by [28]). The three global signals were extracted within the CSF, the WM, and the whole-brain masks. Additionally, a set of physiological regressors were extracted to allow for component-based noise correction [29] (CompCor). Principal components were estimated after high-pass filtering the preprocessed BOLD time-series (using a discrete cosine filter with 128s cut-off) for the two CompCor variants: temporal (tCompCor) and anatomical (aCompCor). tCompCor components were then calculated from the top 5% variable voxels within a mask covering the subcortical regions. This subcortical mask was obtained by heavily eroding the brain mask, which ensured it did not include cortical GM regions. For aCompCor, components were calculated within the intersection of the aforementioned mask and the union of CSF and WM masks calculated in T1w space, after their projection to the native space of each functional run (using the inverse BOLD-to-T1w transformation). Components were also calculated separately within the WM and CSF masks. For each CompCor decomposition, the *k* components with the largest singular values were retained, such that the retained components’ time series were sufficient to explain 50 percent of variance across the nuisance mask (CSF, WM, combined, or temporal). The remaining components were dropped from consideration. The head-motion estimates calculated in the correction step were also placed within the corresponding confounds file. The confound time series derived from head motion estimates and global signals were expanded with the inclusion of temporal derivatives and quadratic terms for each [30]. Frames that exceeded a threshold of 0.5 mm FD or 1.5 standardized DVARS were annotated as motion outliers. All resamplings were performed with *a single interpolation step* by composing all the pertinent transformations (i.e., head-motion transform matrices, susceptibility distortion correction when available, and co-registrations to anatomical and output spaces). Gridded (volumetric) resamplings were performed using antsApplyTransforms (ANTs), configured with Lanczos interpolation to minimize the smoothing effects of other kernels [31]. Non-gridded (surface) resamplings were performed using mri_vol2surf (FreeSurfer, v. 7.1.0; Athinoula A. Martinos Center for Biomedical Imaging, Charlestown, MA, USA).

The resulting preprocessed data from the four runs of each n-back task condition were then deconvolved in a single model. In the deconvolution, signal drift was modeled with a set of basic functions; the motion parameters and their derivatives were used as regressors of no interest, as was framewise displacement. Additionally, the signal from white matter, the ventricles and the global signal, and the derivatives of each of these were included as regressors of no interest. Finally, a regressor representing the onset time of each trial, convolved with a hemodynamic response function was included to model task-related activation. Importantly, the task-related activation was modeled with unit amplitude for all trials across the four runs with the result that only stable, invariant task-related activation was modeled with this regressor. This was done for the 0-back and 2-back conditions separately. The time-varying activation associated with cognitive fatigue was not modeled and was therefore included in the error term. The time-series associated with the error term was saved and used to assess connectivity between areas that have been shown to be related to cognitive fatigue.

**Figure 1:**
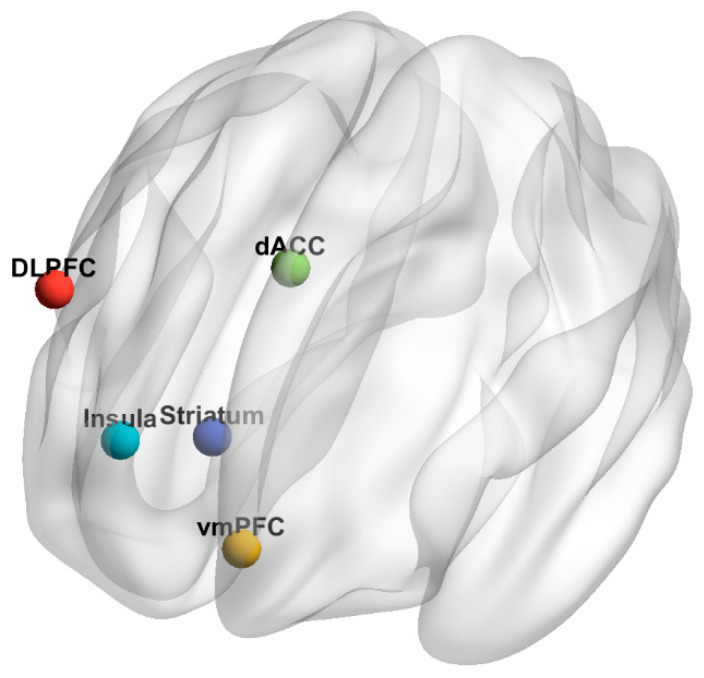
Graphical representation of seed locations. DLPFC, dorsolateral prefrontal cortex (red). vmPFC, ventromedial prefrontal cortex (yellow). dACC, dorsal anterior cingulate cortex (green). Insula (light blue). Striatum (dark blue).

Five seeds were chosen for the analyses, one in each of the following locations: the DLPFC, the vmPFC, the dACC, the insula, and the striatum. Table 1 lists the coordinates of the center of a 4mm sphere used for each seed, and Figure 1 is a graphical representation of the seed locations. All three-dimensional brain figures in this paper were created using BrainNet Viewer v. 1.7 [32]. For each seed, the mean percent signal change was calculated within the seed for each volume of the error term time-series. The correlation between this time-series of means and every voxel in the brain was then computed, before being converted into z-scores with Fisher’s R-to-Z transformation [33]. The resulting z-score maps were entered in the group-level analysis.

### 2.7. Statistical Analysis Plan

Data analyses were conducted using the statistical package R (v. 4.0.2; R Foundation for Statistical Computing, Vienna, Austria).

#### 2.7.1. Demographic Characteristics

Group differences in demographic characteristics were analyzed using independent-samples *T*-tests for continuous variables and Pearson’s chi-squared tests for binary variables.

#### 2.7.2. Behavioral Data

To correlate with the fMRI data, the amount of fatigue for each run was calculated as the mean of the VAS-F scores before and after each run. Because we only wanted to include fMRI signals that reflected cognitive fatigue, we excluded data in which participants reported no fatigue for both before and after each run (i.e., VAS-F = 0). Because the VAS-F scores were skewed, we transformed the scores using the Box Cox method. Transformed VAS-F scores and n-back task performance (accuracy and response time) were analyzed using linear mixed effects models, with restricted maximum likelihood estimation (“lme4” package in R). Degrees of freedom were calculated based on the Satterthwaite’s method (“lmerTest” package in R). Fixed factors included group (MS vs. HC), condition (0-back vs. 2-back), and run (runs 1–4). We also allowed each subject to have their own intercept (random effect). Post hoc analyses for significant interaction terms were performed with the “emmeans” package, using Tukey adjustment for multiple comparisons and the Kenward–Roger method in calculating degrees of freedom.

#### 2.7.3. Neuroimaging Data

The contrast between the two conditions (2-back minus 0-back) was analyzed to isolate fatigue induced by the additional task demand. Changes in residual functional connectivity (part of the error term as specified above) in the 2-back condition relative to the 0-back condition were interpreted as related to fatigue. Separate linear mixed effects models (3dLME from the AFNI suite of processing tools) were used for the data from each seed for each of the HC and MS groups. We chose to examine the functional connectivity patterns by group separately because we wanted to see if there were overlapping as well as differential connectivity patterns. Run (runs 1–4 of each condition) was included as a fixed factor, and subject was included as a random factor (random intercept). The transformed VAS-F scores were mean centered for each group and then included as a quantitative variable in the models. The results of these analyses were corrected for multiple comparisons by using an individual voxel probability threshold of *p* < 0.001 and a cluster threshold of 13 voxels (voxel dimension = 3 × 3 × 3 mm). Monte Carlo simulations, using 3dClustSim (v. AFNI_17.2.16, compile date: 19 September 2017), showed this combination to result in a corrected alpha level of *p* < 0.05.

## 3. Results

The sample consisted of 26 pwMS and 14 HCs. Table 2 summarizes the demographic and disease characteristics. There were no significant differences between the two groups in demographic and disease characteristics (*p* > 0.05).

### 3.1. VAS-F Data

**Figure 2:**
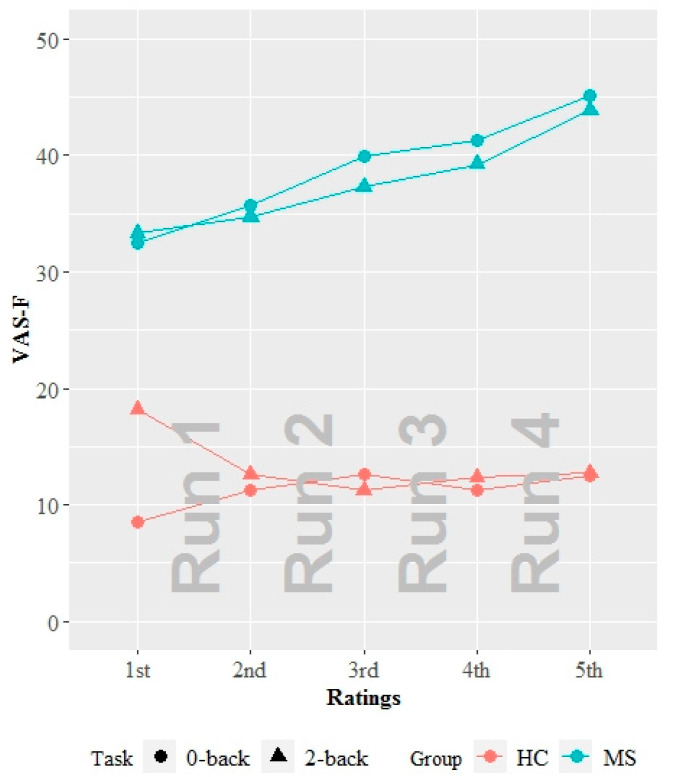
Differences in VAS-F scores. The VAS-F raw scores are plotted as a function of group, condition, and rating. 0-back condition is denoted by circles, and the 2-back condition is denoted by triangles. Red represents the HC group, and turquoise represents the MS group. Abbreviations: VAS-F, Visual Analogue Scale of Fatigue. MS, multiple sclerosis. HC, healthy control.

Using the transformed VAS-F scores, as expected, the MS group reported significantly higher levels of fatigue than the HC group across n-back conditions and runs (estimate = 1.136, standard error (SE) = 0.315, *p* < 0.001; median VAS-F raw scores = 38.27 in MS vs. 12.41 in HC). There was also a significant group × condition interaction (estimate = −0.385, SE = 0.200, *p* = 0.055). Tukey post hoc pairwise comparisons revealed that while there were no differences in fatigue levels between conditions for the HC group (*p* > 0.05), the MS group reported significantly higher levels of fatigue for the 0-back condition than the 2-back condition (estimate = 0.120, SE = 0.043, *p* = 0.028; median VAS-F raw scores = 39.96 in 0-back vs. 37.29 in 2-back). Differences in VAS-F scores were plotted using the raw values to illustrate the actual differences among groups and conditions (Figure 2).

### 3.2. N-Back Task Performance

#### 3.2.1. Accuracy

**Figure 3:**
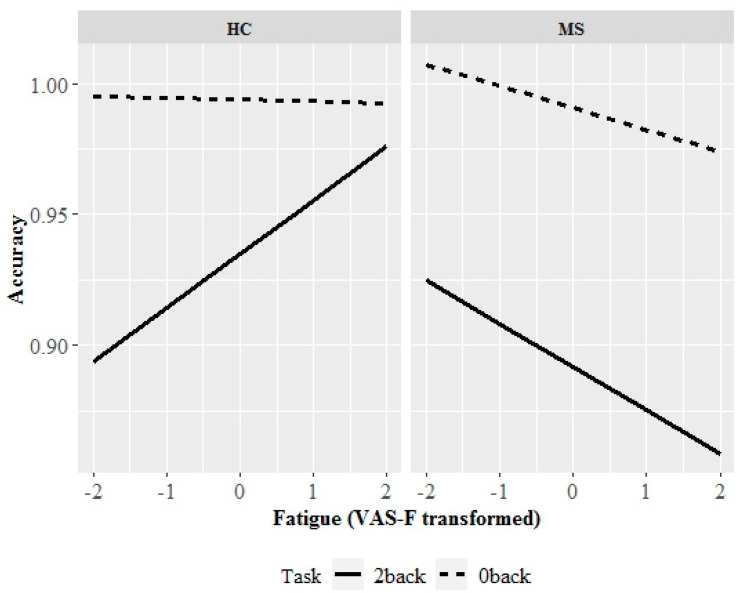
Group × condition × VAS-F (transformed) interaction for n-back task accuracy. For the 2-back condition, there was a positive correlation between fatigue levels and accuracy in the HC group and a negative correlation in the MS group. There were no significant relationships between fatigue levels and accuracy for the 0-back condition. 0-back condition is denoted by dotted lines, and the 2-back condition is denoted by solid lines. Abbreviations: MS, multiple sclerosis. HC, healthy control. VAS-F, Visual Analogue Scale of Fatigue.

There was a significant group × condition × VAS-F (transformed) interaction (estimate = −0.03, SE = 0.01, *p* = 0.028). Tukey post hoc analyses indicated in the HC group, while there was no significant relationship between fatigue levels and accuracy for the 0-back condition, there was a non-statistically significant trend for the 2-back condition such that as fatigue levels increased, accuracy improved (slope = 0.021, confidence intervals [CI] = < −0.001 to 0.042). In the MS group, there was again no significant relationship between fatigue levels and accuracy for the 0-back condition; however, for the 2-back condition, accuracy significantly declined as fatigue levels increased (slope = −0.016; CI = −0.030 to −0.003). The slopes for the 2-back condition were significantly different between the HC and MS groups (estimate = 0.037, SE = 0.013, *p* = 0.023). Figure 3 graphically represents the group × condition × VAS-F (transformed) interaction in task accuracy.

#### 3.2.2. Response Time

Response time was significantly slower for the 2-back condition compared to the 0-back condition for both pwMS and HCs (estimate = 147.88, SE = 27.57, *p* < 0.001), but there were no higher-order interactions for response time.

### 3.3. Neuroimaging Data

#### 3.3.1. DLPFC as Seed

**Figure 4:**
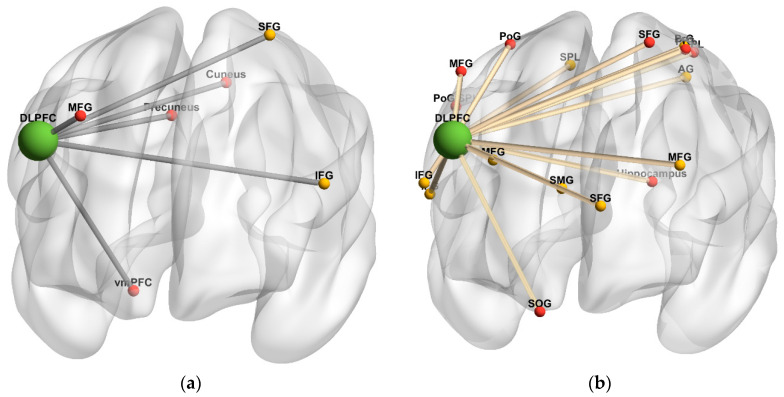
Dorsolateral prefrontal cortex connectivity 3-dimensional rendering. (**a**) Task-independent, fatigue-related connectivity in the healthy control group; (**b**) Task-independent, fatigue-related connectivity in the multiple sclerosis group. The seed is denoted by green spheres; regions with increased connectivity to the seed in the 2-back condition are denoted by yellow spheres, and regions with increased connectivity to the seed in the 0-back condition are denoted by red spheres. Cerebellar regions were omitted. Anterior orientation is in the front of the figure; right hemisphere is on the left side, and left hemisphere is on the right side. Abbreviations: SFG, superior frontal gyrus. MFG, middle frontal gyrus. IFG, inferior frontal gyrus. vmPFC, ventromedial prefrontal cortex. SMG, superior medial gyrus. PrG, precentral gyrus. PoG, postcentral gyrus. SOG, superior orbital gyrus. SPL, superior parietal lobule. AG, angular gyrus.

Table 3 and Figure 4 summarize brain regions with significantly increased task-independent residual functional connectivity with the DLPFC as VAS-F scores increased, in either the 2-back or 0-back condition among the HC and MS groups. In the HC group, as VAS-F scores increased, task-independent residual functional connectivity was significantly increased in the 2-back condition between the DLPFC and left frontal regions (superior and inferior frontal gyri) and significantly increased in the 0-back condition between the DLPFC and right frontal areas (middle frontal gyrus, vmPFC), the right precuneus, and the right cuneus. In the MS group, as VAS-F scores increased, task-independent residual functional connectivity was significantly increased in the 2-back condition between the DLPFC and bilateral frontal regions (superior, middle, and inferior frontal, precentral gyri), the right superior parietal lobule (SPL), and the left angular gyrus, and it significantly increased in the 0-back condition between the DLPFC and bilateral frontal regions (superior, middle, and orbital frontal, precentral gyri), the right postcentral gyrus, the left SPL, the left hippocampus, and the left cerebellum.

#### 3.3.2. vmPFC as Seed

**Figure 5:**
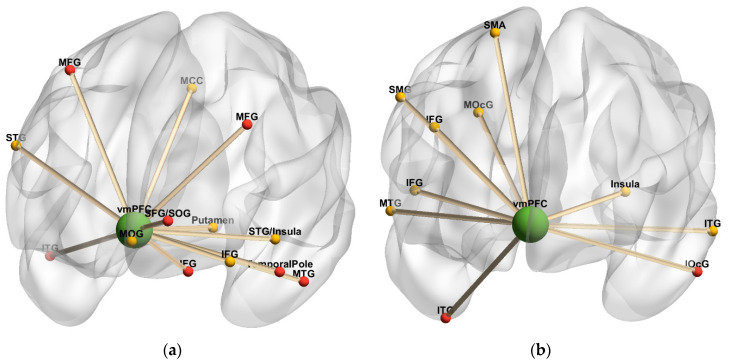
Ventromedial prefrontal cortex connectivity 3-dimensional rendering. (**a**) Task-independent, fatigue-related connectivity in the healthy control group. (**b**) Task-independent, fatigue-related connectivity in the multiple sclerosis group. The seed is denoted by green spheres; regions with increased connectivity to the seed in the 2-back condition are denoted by yellow spheres, and regions with increased connectivity to the seed in the 0-back condition are denoted by red spheres. Cerebellar regions were omitted. Anterior orientation is in the front of the figure; right hemisphere is on the left side, and left hemisphere is on the right side. Abbreviations: SFG, superior frontal gyrus. MFG, middle frontal gyrus. IFG, inferior frontal gyrus. vmPFC, ventromedial prefrontal cortex. MCC, middle cingulate cortex. SMA, supplementary motor area. SOG, superior orbital gyrus. MOG, middle orbital gyrus. SMG, supramarginal gyrus. STG, superior temporal gyrus. MTG, middle temporal gyrus. ITG, inferior temporal gyrus. MOcG, middle occipital gyrus. IOcG, inferior occipital gyrus.

Table 4 and Figure 5 summarize brain regions with significantly increased task-independent residual functional connectivity with the vmPFC as VAS-F scores increased, in either the 2-back or 0-back condition among MS and HC groups. In the HC group, as VAS-F scores increased, task-independent residual functional connectivity was significantly increased in the 2-back condition between the vmPFC and left inferior frontal areas (middle orbital, and inferior), left middle cingulate cortex (MCC), bilateral superior temporal, the left putamen, and the right cerebellum, and it significantly increased in the 0-back condition between the vmPFC and primarily left frontal areas (superior, middle, and inferior) and bilateral temporal areas (middle and inferior temporal gyri, temporal pole). In the MS group, as VAS-F scores increased, task-independent residual functional connectivity was significantly increased in the 2-back condition between the vmPFC and right frontal regions (inferior frontal and supplemental motor areas), the left insula, bilateral temporal regions (middle and inferior), the right supramarginal gyrus, the right middle occipital gyrus, and the left cerebellum, and it significantly increased in the 0-back condition between the vmPFC and the right inferior temporal gyrus and the left inferior occipital gyrus.

#### 3.3.3. dACC as Seed

**Figure 6:**
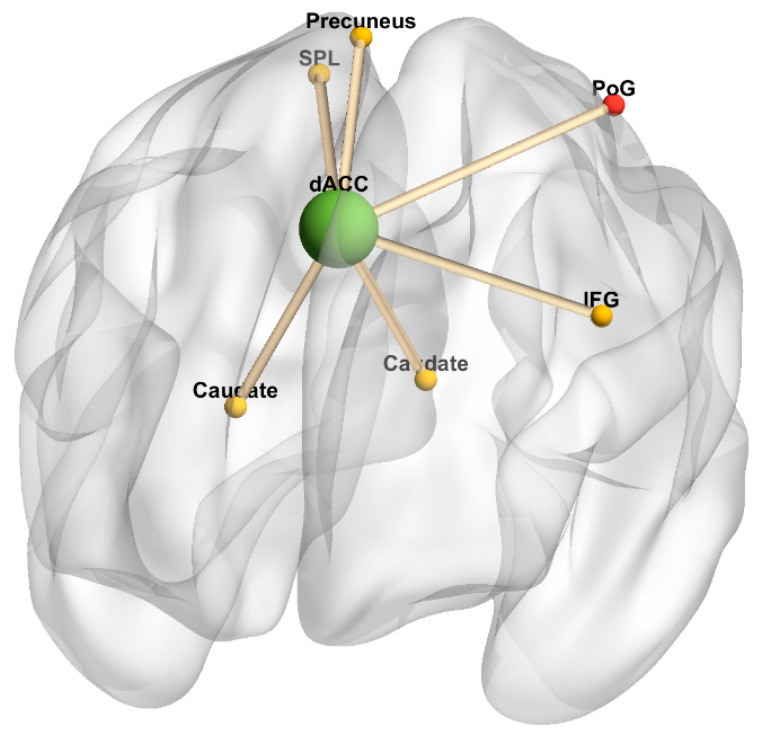
Dorsal anterior cingulate cortex connectivity 3-dimensional rendering. Figure represents task-independent, fatigue-related connectivity in the multiple sclerosis group; there were no significant differences between conditions in the healthy control group. The seed is denoted by a green sphere; regions with increased connectivity to the seed in the 2-back condition are denoted by yellow spheres, and regions with increased connectivity to the seed in the 0-back condition are denoted by red spheres. Cerebellum was omitted. Anterior orientation is in the front of the figure, right hemisphere is on the left side, and left hemisphere is on the right side. Abbreviations: dACC, dorsal anterior cingulate cortex. IFG, inferior frontal gyrus. PoG, postcentral gyrus. SPL, superior parietal lobule.

There were no fatigue-related changes in task-independent residual functional connectivity in the HC group. Table 5 and Figure 6 summarize brain regions with significantly increased task-independent residual functional connectivity with the dACC as VAS-F scores increased, in either the 2-back or 0-back condition in the MS group. In the MS group, as VAS-F scores increased, task-independent residual functional connectivity was significantly increased in the 2-back condition between the dACC and the left inferior frontal gyrus, right parietal regions (SPL, precuneus), and bilateral caudate nuclei, and it significantly increased in the 0-back condition between the dACC and left postcentral gyrus and the right cerebellum.

#### 3.3.4. Insula as Seed

**Figure 7:**
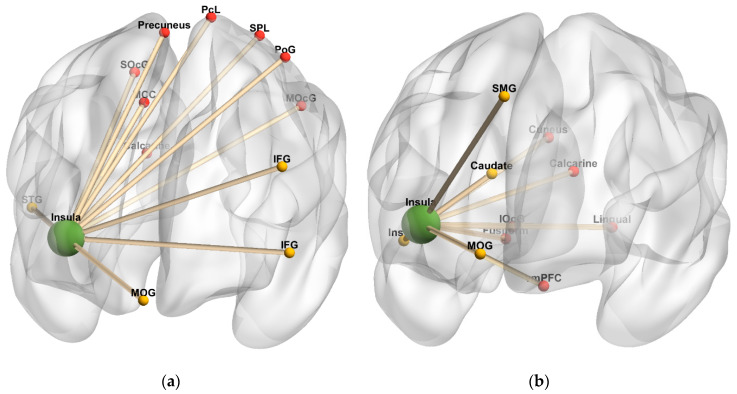
Insula connectivity 3-dimensional rendering. (**a**) Task-independent, fatigue-related connectivity in the healthy control group. (**b**) Task-independent, fatigue-related connectivity in the multiple sclerosis group. The seed is denoted by green spheres; regions with increased connectivity to the seed in the 2-back condition are denoted by yellow spheres, and regions with increased connectivity to the seed in the 0-back condition are denoted by red spheres. Cerebellar regions were omitted. Anterior orientation is in the front of the figure; right hemisphere is on the left side, and left hemisphere is on the right side. Abbreviations: IFG, inferior frontal gyrus. vmPFC, ventromedial prefrontal cortex. SMG, superior medial gyrus. MCC, middle cingulate cortex. PcL, paracentral lobule. PoG, postcentral gyrus. MOG, middle orbital gyrus. STG, superior temporal gyrus. SPL, superior parietal lobule. SOcG, superior occipital gyrus. MOcG, middle occipital gyrus. IOcG, inferior occipital gyrus.

Table 6 and Figure 7 summarize brain regions with significantly increased task-independent residual functional connectivity with the insula as VAS-F scores increased, in either the 2-back or 0-back condition among the MS and HC groups. In the HC group, as VAS-F scores increased, task-independent residual functional connectivity was significantly increased in the 2-back condition between the insula and the left inferior/orbital frontal gyri and the right superior temporal gyrus, and it significantly increased in the 0-back condition between the insula and the right MCC, the left paracentral lobule and postcentral gyrus, the left SPL, the right precuneus, bilateral occipital regions (superior and middle occipital and calcarine), and the right cerebellum. In the MS group, as VAS-F scores increased, task-independent residual functional connectivity was significantly increased in the 2-back condition between the insula and the right middle orbital gyrus, the left superior medial gyrus, the right insula, and the right caudate nucleus, and it significantly increased in the 0-back condition between the insula and the left vmPFC, the right fusiform gyrus, primarily right occipital regions (inferior occipital, calcarine, cuneus, and lingual), and the left cerebellum.

#### 3.3.5. Striatum as Seed

**Figure 8:**
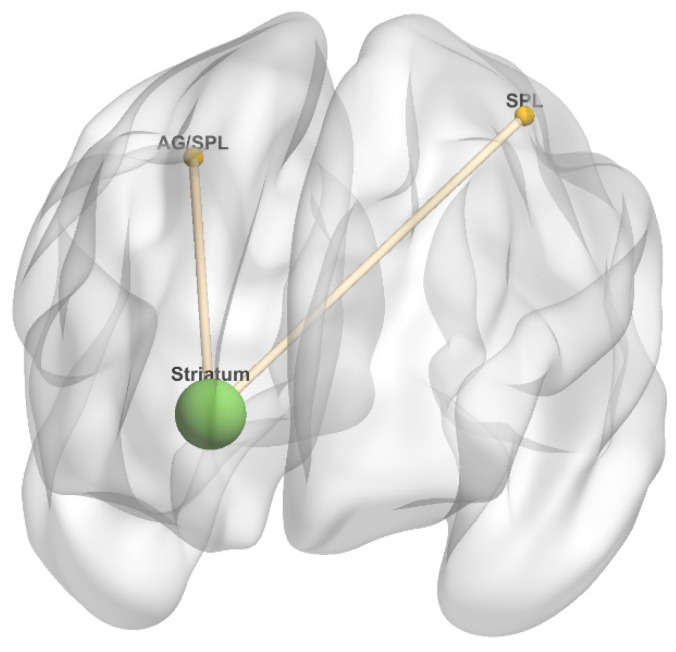
Striatum connectivity 3-dimensional rendering. Figure represents task-independent, fatigue-related connectivity in the multiple sclerosis group; there were no significant differences between conditions in the healthy control group. The seed is denoted by a green sphere, and regions with increased connectivity to the seed in the 2-back condition are denoted by yellow spheres; there were no regions with increased connectivity to the seed in the 0-back condition. Anterior orientation is in the front of the figure; right hemisphere is on the left side, and left hemisphere is on the right side. Abbreviations: SPL, superior parietal lobule. AG, angular gyrus.

There were no fatigue-related changes in task-independent residual functional connectivity in the HC group. Table 7 and Figure 8 summarize brain regions with significantly increased task-independent residual functional connectivity with the striatum as VAS-F scores increased, in either the 2-back or 0-back condition in the MS group. In the MS group, as VAS-F scores increased, task-independent residual functional connectivity was significantly increased in the 2-back condition between the striatum and the left SPL and the right angular gyrus/SPL. There were no regions with increased task-independent residual functional connectivity in the 0-back condition with the striatum in the MS group.

## 4. Discussion

The current study examined cognitive fatigue-related functional connectivity (independent of task-related activations) in a heterogeneous sample of pwMS compared to demographically matched HCs. Results indicated that as state cognitive fatigue increased, HCs showed a left lateralized connectivity pattern and increased connectivity between the striatum of the basal ganglia and the vmPFC (which are crucial in reward processing) during the fatigue-inducing, higher cognitive load condition (2-back) compared to the lower cognitive load control condition (0-back). In contrast, pwMS displayed a more bilateral connectivity pattern and increased connectivity from interoceptive hubs—the insula and the dACC—to the striatum in the 2-back condition relative to the 0-back condition. Taken together, pwMS’s more diffused functional connectivity pattern associated with increased task demands (designed to induce fatigue) may have been inefficient (as evident in their decline in task accuracy), resulting in increased cognitive fatigue and interoceptive efforts to control bodily states (in order to combat fatigue). On the other hand, HCs appeared successful in modulating fatigue (and even improve task accuracy as fatigue level rose) through the reward circuitry.

Our identification of interoceptive mechanisms in MS-related fatigue is consistent with recent evidence of interoceptive deficits and hyperconnectivity of interoceptive hubs (the ACC and the insula) among fatigued pwMS [13]. Specifically, we found that the dACC and the insula were hyperconnected to the striatum, a structure most commonly implicated in fatigue [7,8,9]. These findings suggest that pwMS may have difficulty with regulating their bodily states as fatigue level rises during a taxing activity, which urges them to direct additional neural resources towards monitoring their internal signals. On the contrary, HCs appear to be actively processing the costs and benefits of exerting additional effort through the reward pathway, which facilitates task performance. Our results regarding the reward pathway are somewhat consistent with previous functional connectivity studies, which have identified negative correlations between fatigue measures and frontal-striatal functional connectivity among fatigued pwMS compared to non-fatigued pwMS and HCs [34,35]. In the current study, only HCs showed a positive correlation between fatigue and frontal-striatal functional connectivity, and no such correlation was found in pwMS. The current study extends previous findings by establishing functional connectivity patterns associated with state fatigue during a cognitively demanding task. Previous studies generally correlated resting-state functional connectivity with trait fatigue (e.g., questionnaires asking about fatigue symptoms over the past week or month), which may be more reflective of brain reorganization due to disease pathology, rather than the experience of feeling fatigued. The current study’s analysis of the task-independent signals associated with reports of in-the-moment fatigue during a challenging cognitive task allowed us to characterize in vivo relationships among brain regions, as state fatigue level rose. These results support a pattern of functional disconnection within the reward pathway in MS-related fatigue, which may be used as a target for interventions. Indeed, we previously discovered that progressive resistance exercise training could increase frontal-striatal functional connectivity and reduce the everyday impact of fatigue [36]. Moreover, we have successfully decreased on-task fatigue using a behavioral gambling task aimed to stimulate the frontal-striatal network (with monetary rewards) in pwMS [37].

Present findings of a more diffused pattern of functional connectivity in MS are consistent with the existing literature, which has posited that functional connectivity changes occur as a response to combat structural damage [38,39,40,41]. Such functional reorganization in MS has long been demonstrated with task-based activations. A recurrent finding is that pwMS often recruit bilateral brain regions to support functions typically subserved by more lateralized networks in HCs, such as in a finger tapping task [42,43]. MS fatigue research, specifically, has repeatedly demonstrated bilateral functional recruitment among fatigued pwMS compared to non-fatigued pwMS and HCs (who exhibit a more lateralized pattern of activation) during challenging (therefore fatiguing) cognitive and motor tasks [44,45]. Our results concur with these observations, with pwMS displaying bilateral connectivity while HCs showing primarily left lateralized connectivity during the more mentally challenging condition. Whether such reorganization is adaptive or maladaptive is up for debate in the field [46] and in actuality may depend on the specific context of the situation (e.g., patient demographic and disease characteristics, types of experimental tasks involved). For example, one of our previous papers demonstrated that altered functional connectivity may be compensatory in earlier stages of the disease (maintaining behavioral performance) but may be maladaptive (and does not improve performance) as the disease progresses [38]. The current study supports a maladaptive framework, as changes in functional connectivity were associated with a decline in task performance. It also extends our previous work, which found an association between cognitive fatigue and an inefficient pattern of task-based activations in pwMS relative to HCs [15].

The current study has a few limitations which should be considered when interpreting its findings. First, although the sample size is comparable to many fMRI studies, it is still relatively small for generalization to the overall population. Therefore, future investigations should confirm these findings with a larger sample size. A larger sample size would also allow for analysis into the roles of disease phenotype and duration. Unfortunately, our small sample size and predominance of the relapsing–remitting course of pwMS precluded us from being able to determine if various MS phenotypes would show a similar connectivity pattern. Second, since the MS group reported much higher levels of fatigue than the HC group overall and started out reporting higher fatigue (first rating), it is unclear whether our observations reflected pathological fatigue in MS or higher levels of fatigue in general. Future research may consider including patient groups of varying pathologies and specific HC groups with high levels of fatigue (e.g., medical residents with long work hours) to test this question. For example, a recent study identified functional connectivity in the insula and the putamen (part of the striatum) as important hubs for cognitive fatigue across multiple cognitive tasks among healthy older adults [10]. Therefore, it is possible that our observations reflect a general fatigue network rather than a MS-specific fatigue network.

## 5. Conclusions

The current study identified altered cognitive fatigue-related functional connectivity in the interoceptive and reward pathways among pwMS. Specifically, pwMS showed a hyperconnectivity within the interoceptive network and disconnection within the reward circuitry. Such alterations may be the result of inefficient brain connectivity when meeting increased task demands.

## Figures and Tables

**Table 1:** Coordinates of center location of seed regions.

Location	X	Y	Z
DLPFC	44	32	36
vmPFC	−6	46	−6
dACC	−4	20	46
Insula	34	22	0
Striatum	18	12	0

Abbreviations: DLPFC, dorsolateral prefrontal cortex. vmPFC, ventromedial prefrontal cortex. dACC, dorsal anterior cingulate cortex.

**Table 2:** Demographic and disease characteristics.

Characteristic	MS (*n* = 26)		HC (*n* = 14)		MS vs. HC (*p*)
	Mean (SD)	Range	Mean (SD)	Range	
Age: years	48.12 (10.09)	26–63	42 (12.87)	24–58	n.s.
Education: years	16.19 (2.19)	12–21	16.31 (1.97)	12–20	n.s.
Illness duration: years	11.05 (6.21)	1–28	-	-	-
	**Number (%)**		**Number (%)**		
Females	23 (88)	-	13 (93)	-	n.s.
MS phenotype	-	-	-	-	-
Relapsing-remitting	19 (73)	-	-	-	-
Primary progressive	2 (8)	-	-	-	-
Secondary progressive	3 (12)	-	-	-	-
Unknown	2 (7)	-	-	-	-

Column named “MS vs. HC (*p*)” denotes group comparisons in demographic variables using independent-samples *T*-tests and Pearson’s chi-squared tests; *p* values are listed for significant variables; non-significant variables are denoted as “n.s.” Abbreviations: MS, multiple sclerosis. HC, healthy control. SD, standard error.

**Table 3:** Task-independent (residual) fatigue-related functional connectivity with the dorsolateral prefrontal cortex as seed.

Dorsolateral Prefrontal Cortex Seed					
Location	X	Y	Z	Voxels	Z Stat
HC					
Increased Task-Independent Residual Connectivity in 2-back Condition					
Superior Frontal Gyrus	−30.7	−4.8	70	19	4.90
Inferior Frontal Gyrus	−54.7	22.7	22	103	4.96
Increased Task-Independent Residual Connectivity in 0-back Condition					
Middle Frontal Gyrus	27.8	39.9	46	27	−4.77
Ventromedial Prefrontal Cortex	14.0	12.4	−18	30	−5.13
Precuneus	14.0	−63.2	34	36	−4.30
Cuneus	0.3	−94.2	42	13	−5.05
MS					
Increased Task-Independent Residual Connectivity in 2-back Condition					
Superior Frontal Gyrus	−16.9	70.8	18	14	3.77
Middle Frontal Gyrus	27.8	39.9	30	14	4.72
Middle Frontal Gyrus	−41.0	43.3	30	29	5.56
Inferior Frontal Gyrus	62.1	12.4	14	16	3.91
Inferior Frontal Gyrus	58.7	39.9	10	25	4.72
Superior Medial Gyrus	0.3	53.7	22	61	6.21
Precentral Gyrus	38.1	2.1	34	15	5.00
Superior Parietal Lobule	20.9	−80.4	50	35	5.80
Angular Gyrus	−37.6	−70.1	54	18	4.66
Increased Task-Independent Residual Connectivity in 0-back Condition					
Superior Frontal Gyrus	−23.8	9.0	70	54	−6.26
Middle Frontal Gyrus	45.0	9.0	58	15	−4.36
Superior Orbital Gyrus	7.1	60.5	−22	24	−4.73
Precentral Gyrus	−41.0	−11.7	70	13	−4.59
Postcentral Gyrus	34.6	−28.9	78	21	−4.43
Postcentral Gyrus/Superior Parietal Lobule	45.0	−42.6	66	42	−4.53
Superior Parietal Lobule	−37.6	−70.1	66	14	−6.01
Hippocampus	−16.9	−35.7	14	15	−4.75
Cerebellum Crus I	−51.3	−49.5	−38	45	−6.36
Cerebellum Crus I	−37.6	−90.7	−30	15	−4.51

“X”, “Y”, and “Z” denote the three-dimensional peak coordinates of each significant clusters. Positive “X” values represent the right hemisphere, and negative “X” values represent the left hemisphere. “Voxels” denote the number of voxels in each cluster. “Z Stat” reflects the extent of functional connectivity between the seed region and the brain region in each row; positive values represent increased functional connectivity in the 2-back condition, and negative values represent increased functional connectivity in the 0-back condition. Abbreviations: MS, multiple sclerosis. HC, healthy control.

**Table 4:** Task-independent (residual) fatigue-related functional connectivity with the ventromedial prefrontal cortex as seed.

Ventromedial Prefrontal Cortex Seed					
Location	X	Y	Z	Voxels	Z Stat
HC					
Increased Task-independent Residual Connectivity in 2-back Condition					
Middle Orbital Gyrus	−10.1	60.5	−10	110	5.45
Inferior Frontal Gyrus	−41.0	36.5	−18	68	5.45
Middle Cingulate Cortex	−3.2	−25.4	46	30	4.84
Superior Temporal Gyrus/Insula	−47.9	5.5	−10	21	4.78
Superior Temporal Gyrus	69.0	−28.9	22	20	4.72
Putamen	−20.4	−1.4	−6	16	5.11
Cerebellar Vermis	7.1	−77.0	−18	20	5.29
Increased Task-independent Residual Connectivity in 0-back Condition					
Superior Frontal Gyrus/Superior Orbital Gyrus	−27.2	67.4	−2	19	−5.10
Middle Frontal Gyrus	−47.9	36.5	34	208	−6.12
Middle Frontal Gyrus	34.6	15.8	62	18	−4.64
Inferior Frontal Gyrus	−20.4	26.1	−22	24	−4.32
Temporal Pole	−54.7	19.3	−22	22	−4.26
Middle Temporal Gyrus	−58.2	2.1	−26	27	−4.06
Inferior Temporal Gyrus	58.7	−39.2	−18	19	−4.00
MS					
Increased Task-independent Residual Connectivity in 2-back Condition					
Inferior Frontal Gyrus	45.0	12.4	6	40	5.20
Inferior Frontal Gyrus	38.1	9.0	30	21	4.42
Supplemental Motor Area	14.0	12.4	66	39	5.10
Insula	−37.6	19.3	6	13	4.43
Middle Temporal Gyrus	65.6	−49.5	2	31	4.14
Inferior Temporal Gyrus	−61.6	−56.4	−6	13	4.15
Supramarginal Gyrus	58.7	−25.4	46	31	4.30
Middle Occipital Gyrus	34.6	−63.2	34	53	5.42
Cerebellum Crus I	−51.3	−49.5	−30	37	5.38
Increased Task-independent Residual Connectivity in 0-back Condition					
Inferior Temporal Gyrus	31.2	2.1	−50	20	−5.51
Inferior Occipital Gyrus	−54.7	−73.6	−18	21	−4.66

“X”, “Y”, and “Z” denote the three-dimensional peak coordinates of each significant clusters. Positive “X” values represent the right hemisphere, and negative “X” values represent the left hemisphere. “Voxels” denote the number of voxels in each cluster. “Z Stat” reflects the extent of functional connectivity between the seed region and the brain region in each row; positive values represent increased functional connectivity in the 2-back condition and negative values represent increased functional connectivity in the 0-back condition. Abbreviations: MS, multiple sclerosis. HC, healthy control.

**Table 5:** Task-independent (residual) fatigue-related functional connectivity with the dorsal anterior cingulate cortex as seed.

Dorsal Anterior Cingulate Cortex Seed					
Location	X	Y	Z	Voxels	Z Stat
MS					
Increased Task-independent Residual Connectivity in 2-back Condition					
Inferior Frontal Gyrus	−47.9	5.5	30	50	4.64
Superior Parietal Lobule	24.3	−73.6	58	43	4.82
Precuneus	7.1	−63.2	66	30	4.63
Caudate Nucleus	−16.9	9.0	18	24	4.14
Caudate Nucleus	14.0	22.7	14	18	4.38
Increased Task-independent Residual Connectivity in 0-back Condition					
Postcentral Gyrus	−41.0	−28.9	62	46	−5.34
Cerebellum Crus 1	41.5	−83.9	−34	16	−4.98

“X”, “Y”, and “Z” denote the three-dimensional peak coordinates of each significant clusters. Positive “X” values represent the right hemisphere, and negative “X” values represent the left hemisphere. “Voxels” denote the number of voxels in each cluster. “Z Stat” reflects the extent of functional connectivity between the seed region and the brain region in each row; positive values represent increased functional connectivity in the 2-back condition and negative values represent increased functional connectivity in the 0-back condition. Abbreviations: MS, multiple sclerosis. HC, healthy control.

**Table 6:** Task-independent (residual) fatigue-related functional connectivity with the insula as seed.

Insula Seed					
Location	X	Y	Z	Voxels	Z Stat
HC					
Increased Task-independent Residual Connectivity in 2-back Condition					
Inferior Frontal Gyrus	−51.3	26.1	−2	51	5.45
Inferior Frontal Gyrus	−47.9	22.7	30	18	3.81
Middle Orbital Gyrus	−6.6	70.8	−14	15	4.18
Superior Temporal Gyrus	55.3	−25.4	2	18	4.96
Increased Task-independent Residual Connectivity in 0-back Condition					
Middle Cingulate Cortex	14.0	−32.3	42	20	−4.47
Paracentral Lobule	−10.1	−39.2	82	13	−5.33
Postcentral Gyrus	−41.0	−46.1	66	17	−4.84
Superior Parietal Lobule	−30.7	−42.6	74	22	−4.46
Precuneus	7.1	−63.2	66	51	−4.60
Superior Occipital Gyrus	24.3	−70.1	46	16	−3.95
Middle Occipital Gyrus	−37.6	−77.0	34	34	5.46
Calcarine Gyrus	20.9	−77.0	14	13	−4.60
Cerebellum lobule VIII	24.3	−35.7	−50	22	−4.74
MS					
Increased Task-independent Residual Connectivity in 2-back Condition					
Middle Orbital Gyrus	0.3	64.0	−10	68	5.09
Superior Medial Gyrus	−6.6	57.1	46	21	5.11
Insula	45.0	5.5	−6	26	4.24
Caudate Nucleus	10.6	12.4	18	13	5.91
Increased Task-independent Residual Connectivity in 0-back Condition					
Ventromedial Prefrontal Cortex	−6.6	5.5	−22	15	−6.21
Fusiform Gyrus	27.8	−66.7	−6	13	−4.09
Inferior Occipital Gyrus	31.2	−87.3	−2	19	−3.70
Calcarine Gyrus	3.7	−70.1	18	15	−4.03
Cuneus	14.0	−73.6	30	26	−5.39
Lingual Gyrus	−6.6	−83.9	−2	39	−4.88
Cerebellum Crus I	−54.7	−56.4	−38	15	−4.62
Cerebellum Lobule VI	−20.4	−66.7	−14	14	−4.85
Cerebellum Lobule VIII	−16.9	−63.2	−62	26	−4.46

“X”, “Y”, and “Z” denote the three-dimensional peak coordinates of each significant clusters. Positive “X” values represent the right hemisphere, and negative “X” values represent the left hemisphere. “Voxels” denote the number of voxels in each cluster. “Z Stat” reflects the extent of functional connectivity between the seed region and the brain region in each row; positive values represent increased functional connectivity in the 2-back condition, and negative values represent increased functional connectivity in the 0-back condition. Abbreviations: MS, multiple sclerosis. HC, healthy control.

**Table 7:** Task-independent (residual) fatigue-related functional connectivity with the striatum as seed.

Striatum Seed					
Location	X	Y	Z	Voxels	Z Stat
MS					
Increased Task-independent Residual Connectivity in 2-back Condition					
Superior Parietal Lobule	−30.7	−80.4	58	16	4.73
Angular Gyrus/Superior Parietal Lobule	41.5	−80.4	42	16	4.58

“X”, “Y”, and “Z” denote the three-dimensional peak coordinates of each significant clusters. Positive “X” values represent the right hemisphere, and negative “X” values represent the left hemisphere. “Voxels” denote the number of voxels in each cluster. “Z Stat” reflects the extent of functional connectivity between the seed region and the brain region in each row; positive values represent increased functional connectivity in the 2-back condition, and negative values represent increased functional connectivity in the 0-back condition. Abbreviations: MS, multiple sclerosis. HC, healthy control.
